# Type and Extent of Somatic Morbidity before and after the Diagnosis of Hypothyroidism. A Nationwide Register Study

**DOI:** 10.1371/journal.pone.0075789

**Published:** 2013-09-16

**Authors:** Marianne Thvilum, Frans Brandt, Dorthe Almind, Kaare Christensen, Thomas Heiberg Brix, Laszlo Hegedüs

**Affiliations:** 1 Department of Endocrinology and Metabolism, Odense University Hospital, Odense C, Denmark; 2 The Danish Aging Research Center and The Danish Twin Registry, University of Southern Denmark, Odense C, Denmark; 3 Department of Clinical Genetics, Odense University Hospital, Odense C, Denmark; 4 Department of Clinical Biochemistry and Pharmacology, Odense University Hospital, Odense C, Denmark; IPATIMUP/Faculty of Medicine of the University of Porto, Portugal

## Abstract

**Background:**

Hypothyroidism has been linked with an increased risk of other morbidities, such as cardiovascular diseases and diabetes mellitus. However, the temporal relationship between these diseases and the diagnosis of hypothyroidism is not well illuminated. Such information may provide insight into causal relationships between hypothyroidism and other morbidities.

**Aim:**

To investigate the type and extent of somatic morbidity before and after a diagnosis of hypothyroidism.

**Methods:**

Observational cohort study. From official Danish health registers, 2822 hypothyroid singletons were identified and matched 1:4 with non-hypothyroid controls and observed over a mean period of 6 years. Frequency of different morbidities was obtained by person-to-person linking in the registers. Logistic and Cox regression models were used to assess the risk of morbidity before and after the diagnosis of hypothyroidism, respectively.

**Results:**

Prior to the diagnosis of hypothyroidism there was a significantly increased risk of being diagnosed with cardiovascular diseases (odds ratio (OR) 1.37; 95% confidence interval (CI): 1.19–1.58), lung diseases (OR 1.25; 95% CI: 1.13–1.39), diabetes mellitus (OR 1.92; 95% CI: 1.61–2.29), as well as malignant diseases (OR 1.24; 95% CI: 1.06–1.45). Following the diagnosis of hypothyroidism there was a significantly increased risk of being diagnosed with cardiovascular diseases (hazard ratio (HR) 1.36; 95% CI: 1.15–1.60); lung diseases (HR 1.51; 95% CI: 1.30–1.75); and diabetes mellitus (HR 1.40; 95% CI: 1.11–1.77).

**Conclusions:**

Prior to the diagnosis of hypothyroidism there is an excess risk of being diagnosed with cardiovascular diseases, lung diseases, diabetes mellitus, and malignant diseases. Following the diagnosis of hypothyroidism we demonstrate an increased frequency of cardiovascular diseases, lung diseases, and diabetes mellitus.

## Introduction

Hypothyroidism is a common endocrine disorder, with a prevalence of 1–2% [1]. The development of hypothyroidism is influenced by a number of factors such as: gender [1], genetic predisposition [2], and environmental contributors like smoking [3]–[5]. It is well accepted that the cardiovascular system is a major target of thyroid hormone action. The most frequent changes in cardiac hemodynamics, related to hypothyroidism, are increased systemic vascular resistance, decreased cardiac preload, as well as reduced diastolic and systolic function [6]–[8]. Accordingly, hypothyroidism has been associated with symptoms of cardiac dysfunction, such as reduced exercise tolerance and dyspnea on effort [9], [10], as well as overt cardiovascular diseases [11], [12]. Hypothyroidism has also been linked with an increased frequency of a variety of other conditions, such as lung diseases [13], diabetes mellitus [14], [15], and rheumatic diseases [16]. However, due to small sample sizes and lack of considering potential confounding and temporality, the association between hypothyroidism and other morbidities still remains to be clarified [16]. Despite this, our recent studies provide robust evidence for an association between thyroid dysfunction, hypo- [17], [18] as well as hyperthyroidism [19]–[21], and an overall increased mortality.

In the present study, we used Danish nationwide registers to evaluate whether hypothyroidism is associated with various morbidities. By investigating incident hypothyroid cases, in a prospective manner, we were able to evaluate temporality of cardiovascular diseases, lung diseases, diabetes mellitus, rheumatic diseases and malignant diseases in relation to hypothyroidism. In order to control for possible genetic confounding [22], we evaluated the extent of disease in twin pairs discordant for hypothyroidism.

## Materials and Methods

### Data sources

The Danish Civil Registration System (DCRS) and The Danish Demographic Database (DDB) cover information on demographics, vital status, date of death and residence of all persons living or having lived in Denmark from 1968 [23]. We have identified a random 5% sample of the Danish background population covering the birth cohorts 1870–2001 from DCRS.

The Danish Twin Registry (DTR) is a nationwide and population-based register, which was established in 1954 and comprises nearly 150,000 twins born in Denmark between 1870 and 2001 [24]. All twin pairs are ascertained independently of zygosity and disease.

The Danish National Patient Registry (DNPR) includes registrations of all admissions to hospitals since January 1, 1977. Outpatient admissions have been registered separately since January 1, 1995 [23]. All registrations are according to the International Classification of Disease (ICD). The validity of the DNPR is high and misclassification of thyroid dysfunction has been shown to occur in less than 2% of cases [25].

The Danish National Prescription Registry (DNPrR) provides information on all prescriptions of drugs dispensed from Danish pharmacies since 1995 [23]. Coding for medical products is according to the Anatomical Therapeutic Chemical (ATC) classification system. Besides the ATC code the register covers information on date of dispensing, strength, and quantity (in defined daily doses). In Denmark, the national health security system covers all inhabitants and partially reimburses drug expenses. Data from the DNPrR are transmitted directly from the cash register in the pharmacy and used in the calculation (made on an individual level) of the expenses reimbursed.

All described databases are hosted at Statistics Denmark [23]. DCRS is based on a unique 10 digit personal identification number (CPR-number) assigned to all persons living or having lived in Denmark. The CPR-number allows record-linkage between all the mentioned databases on an individual level.

### Diagnosis of hypothyroidism

Information on thyroid status was drawn from DNPR and DNPrR. To be classified as having hypothyroidism, subjects should be recorded in at least one of these registers. In DNPR hypothyroidism was defined by ICD-10 codes (E03.2-E03.9). In DNPrR hypothyroidism was defined by at least two dispensed prescriptions of thyroid hormone (ATC = H03A). Subjects with primary hypothyroidism diagnosed after the age of 18 were eligible for the present study. Thus, individuals diagnosed with malignant thyroid diseases, congenital hypothyroidism or pituitary hypothyroidism, represented by the ICD-8 codes 193.99, 243.99, 253.00–253.02, 253.09–253.11, 253.15, 253.18, 253.19, 253.90, 253.99 and the ICD-10 codes C73, E00.0-E00.9, E03.0-E03.1, E22.0-E22.9, E23.0-E23.7 and E24.0, were excluded. Furthermore, cases diagnosed with hyperthyroidism (defined by the ICD-8 codes: 242.00 to 242.09 and the ICD-10: E05.0 to E05.9 in DNPR or having received dispensed prescriptions of antithyroid drugs, ATC = H03B in DNPrR, prior to the diagnosis of hypothyroidism, were excluded.

### Study population

The study populations, singletons and twins, were identified from DCRS [23] and DTR [24], respectively. In order to investigate incident cases - and to obtain the same observation frame in DCRS, DTR, DNPR, and DNPrR - only individuals diagnosed with hypothyroidism after December 31, 1995 were included. In all, 2822 singletons with hypothyroidism were matched 1:4 according to age and gender with 11288 non-hypothyroid controls, using the principles of density sampling [26]. From the DTR, 385 same sexed twin pairs discordant for hypothyroidism were identified. All participants were followed until migration, death, or December 31, 2008, whichever came first.

### Morbidity

The Charlson score accounts for 19 disease groups (myocardial infarction, heart failure, vascular disease, cerebrovascular disease, dementia, chronic lung disease, rheumatic disease, gastric ulcer, liver disease, diabetes mellitus without complications, diabetes mellitus with complications, hemiplegia, kidney disease, cancer, cancer with metastases, lymphoma, leukemia, liver failure, and AIDS) by creating a weighted score on an individual level, to optimize the prediction of the one-year mortality risk within each disease category [27]. The Charlson score was originally constructed to estimate one-year mortality in patients with breast cancer, but has subsequently been validated and used in different phenotypes, including non-malignant diseases [28]. All the categories included in the Charlson score were identified from DNPR, and for diabetes mellitus as well as lung diseases also from DNPrR. As patients with diabetes and obstructive airway diseases are often diagnosed and treated solely in primary care, all users of anti-diabetics (ATC: A10) and drugs for obstructive airway diseases (ATC: R03) identified from DNPrR [23] were categorized with diabetes and lung diseases, respectively. All relevant ATC-codes as well as ICD-8 and ICD-10 codes are shown in Table 1.

**Table 1:** Identification of morbidity in the Danish National Patient Registry and the Danish National Prescription Registry.

Morbidity - outcome	ICD-8^a^	ICD-10^b^	ATC^c^
**Cardiovascular diseases**	344, 410, 427, 428, 430–438, 440–445 and 782	I1, I2, I5–I7, G4 and G8	_
**Lung diseases**	490–493 and 515–518	J4, J6, J8 and J9	RO3
**Diabetes mellitus**	249 and 250	E1	A10
**Rheumatic diseases**	135, 446, 712 and 734	O8, M0 and M3	_
**Malignant diseases**	140–163, 170–174, 180–199 and 200–207	C0–C9	_
**Other diseases^d^**	290, 293, 530–534, 571, 573, 070, 456, 344, 403–404, 580–584, 590, 593, 753, 792, and 079	F0, G3, K2, K7, B1, G8, I1, N0–N1, Q6, and B2	_

a) International Classification of Diseases 8.

b) International Classification of Diseases 10.

c) Anatomical Therapeutic Chemical classification system.

d) Dementia, gastric ulcer, liver disease, liver failure, hemiplegia, kidney disease, and AIDS.

The Charlson score, prior to the diagnosis of hypothyroidism, was used in the adjustments of analyses of the risk of morbidity following the diagnosis of hypothyroidism. For subjects with hypothyroidism, the Charlson score reflects the time period from January 1, 1977 (start of DNPR) until the index-date (date of the diagnosis of hypothyroidism). In controls, the Charlson score covers the time period from the start of DNPR until the index-date of the corresponding case.

The overall outcomes, categorized into cardiovascular diseases, lung diseases, diabetes mellitus, rheumatic diseases, malignant diseases, and other diseases were defined on the basis of the 19 categories covered by the Charlson score (Table 1).

### Data Analyses

Cohort frequencies were compared with the Pearson X^2^ test, whereas group means and medians were compared by a t-test. In the case of paired comparisons the paired t-test was used.

In the singleton population, morbidity prior to the diagnosis of hypothyroidism was evaluated in a logistic regression analysis adjusted for age and sex. Following the diagnosis of hypothyroidism the relationship between hypothyroidism and morbidity was evaluated by a Cox regression model. Age was chosen as the underlying time variable. In both cases and controls, person years of follow-up were accumulated from the index-date of the case and terminated on the date of diagnosis of morbidity, migration, death, or end of follow-up (December 31, 2008), whichever came first.

In all Cox analyses of the singletons the variable “pair” was used as a stratum variable, fixing the baseline hazard within a matched pair, while at the same time allowing this baseline hazard to vary freely between pairs. Subsequently, using the Charlson score, all Cox regression analyses were adjusted for the degree of co-morbidity preceding the diagnosis of hypothyroidism.

In order to evaluate potential detection bias (Berkson’s bias) [29], all analyses were repeated with a censoring of diagnoses made within 365 days before and after the diagnosis of hypothyroidism.

Additionally, we performed intra-pair analysis of the twin population. Here, the hypothyroid twin was matched with the corresponding euthyroid co-twin. After pooling all disease groups, the differences in overall frequency of diseases among the twin pairs discordant for hypothyroidism were evaluated by a conditional logistic regression analysis.

Significant differences were defined as a p-value below 0.05, using two-tailed tests. All analyses were conducted using STATA version 11.0 (2009; Stata Corporation, College Station, TX, USA).

## Results

### Characteristics of the study populations

Characteristics of the 2822 singletons diagnosed with hypothyroidism, as well as the 385 twin pairs discordant for hypothyroidism are presented in Table 2. There were more females than males with hypothyroidism and the mean age at diagnosis of singletons and twins were 58 and 54 years, respectively. All cases as well as controls were observed over a mean period of 6 years.

**Table 2:** Characteristics of the study populations.

	Cases, singletons	Controls, singletons	Hypothyroid twins^a^	Euthyroid twins^a^
**Number**	2822	11288	385	385
**Mean age**	58^b^	58	54^b^	54
**Females (%)**	84	84	82	82
**Mean follow-up (years)**	6	6	6	6

a) From twin pairs discordant for hypothyroidism.

b) Mean age at diagnosis of hypothyroidism.

### Overall associations between hypothyroidism and morbidity

The frequency of cardiovascular diseases was higher in subjects with hypothyroidism than in controls (19% versus 15%, p<0.001) (Table 3). This was also true for lung diseases, diabetes mellitus, malignant diseases, and the group of other diseases. In contrast, there was no increased risk of being diagnosed with rheumatic diseases.

**Table 3:** Temporality and impact of morbidity in singletons diagnosed with hypothyroidism.

Disease category	Cases^a^	Controls^a^	P-value^b^	Odds ratio^c^	Hazard ratio^d^
**Cardiovascular diseases**	**543 (19%)**	**1685 (15%)**	**<0.001**	**1.37 (1.19**–**1.58)**	**1.36 (1.15**–**1.60)**
**Lung diseases**	**807 (29%)**	**2543 (23%)**	**<0.001**	**1.25 (1.13**–**1.39)**	**1.51 (1.30**–**1.75)**
**Diabetes mellitus**	**299 (11%)**	**721 (6%)**	**<0.001**	**1.92 (1.61**–**2.29)**	**1.40 (1.11**–**1.77)**
**Rheumatic diseases**	109 (4%)	387 (3%)	0.263	1.04 (0.81–1.35)	1.37 (0.91–2.07)
**Malignant diseases**	**359 (13%)**	**1252 (11%)**	**0.015**	**1.24 (1.06**–**1.45)**	1.00 (0.81–1.24)
**Other diseases^e^**	**296 (10%)**	**917 (8%)**	**<0.001**	1.13 (0.92–1.39)	1.27 (0.99–1.65)

a) Overall associations: number of individuals with a first time hit of the respective disease category.

b) P-value from overall associations.

c) Odds ratios before the diagnosis of hypothyroidism, adjusted for age and sex.

d) Hazard ratios after the diagnosis of hypothyroidism, adjusted for the Charlson score.

e) Dementia, gastric ulcer, liver disease, liver failure, hemiplegia, kidney disease, and AIDS Data given in bold represent significant findings.

### Morbidity preceding the diagnosis of hypothyroidism

As evident from Table 3, subjects with hypothyroidism had a significantly increased risk of being diagnosed with cardiovascular diseases, lung diseases, diabetes mellitus as well as malignant diseases prior to the diagnosis of hypothyroidism; odds ratio (OR)_cardiovascular diseases_ 1.37; 95% confidence interval (CI): 1.19–1.58, OR_lung diseases_ 1.25; 95% CI: 1.13–1.39, OR_diabetes mellitus_ 1.92; 95% CI: 1.61–2.29, and OR_malignant diseases_ 1.24; 95% CI: 1.06–1.45. Rheumatic diseases and the group of other diseases were not significantly increased before the diagnosis of hypothyroidism. Evaluating the same disease categories, but censoring diagnoses made within 365 days prior to the diagnosis of hypothyroidism, yielded essentially similar results: OR_cardiovascular diseases_ 1.30; 95% CI: 1.12–1.50, OR_lung diseases_ 1.19; 95% CI: 1.06–1.32, OR_diabetes mellitus_ 1.66; 95% CI: 1.38–2.01, OR_rheumatic diseases_ 1.03; 95% CI: 0.79–1.35, OR_malignant diseases_ 1.21; 95% CI: 1.03–1.42, and OR_other diseases_ 1.03; 95% CI: 0.83–1.28. When performing the above analyses in hypothyroid patients identified solely from DNPrR, the results attenuated, but did not change significantly (data not shown).

### Morbidity following the diagnosis of hypothyroidism

After the diagnosis of hypothyroidism there was a statistically increased risk of being diagnosed with cardiovascular diseases (hazard ratio (HR) 1.36; 95% CI: 1.15–1.60), lung diseases (HR 1.51; 95% CI: 1.30–1.75), and diabetes mellitus (HR 1.40; 95% CI: 1.11–1.77) (Table 3). The frequency of rheumatic diseases, malignant diseases, and the group of other diseases were not significantly increased after the diagnosis of hypothyroidism. These results did not change significantly, when applying censoring of diagnoses made within 365 days following the diagnosis of hypothyroidism: HR_cardiovascular diseases_ 1.04; 95% CI: 0.85–1.27, HR_lung diseases_ 1.42; 95% CI: 1.20–1.68, HR_diabetes mellitus_ 1.33; 95% CI: 1.02–1.74, HR_rheumatic diseases_ 1.16; 95% CI: 0.71–1.88, HR_malignant diseases_ 1.05; 95% CI: 0.92–1.19, and HR_other diseases_ 1.17; 95% CI: 0.87–1.59. The results attenuated, but did not change significantly when performing the above analyses in hypothyroid patients identified solely from DNPrR (data not shown).

### Overall associations between hypothyroidism and morbidity in twins

In twin pairs discordant for hypothyroidism, the frequency of morbidity was significantly higher in the hypothyroid twins compared to their non-hypothyroid co-twins (OR 1.42; 95% CI: 1.04–1.94). When stratifying for zygosity, the estimates remained increased but were no longer significant (dizygotic twins: OR 1.32; 95% CI: 0.90–1.93, and monozygotic twins: OR 1.65; 95% CI: 0.95–2.88). For the same reason, lack of power, subdividing the twin data according to morbidity before and after the diagnosis of hypothyroidism, and according to type of morbidity, was not meaningful.

## Discussion

While we recently demonstrated a 38% excess mortality in hypothyroidism [18], the type and extent of the underlying morbidities remain to be clearly defined. In the present study, based on population-based Danish registers, we found that hypothyroidism was positively associated with cardiovascular diseases, lung diseases, diabetes mellitus and malignant diseases. When analyzing the temporal relationship between hypothyroidism and morbidity, we found an increased burden of cardiovascular diseases, lung diseases and diabetes mellitus both before and after the diagnosis of hypothyroidism.

Our findings of an overall positive association between hypothyroidism and cardiovascular diseases, lung diseases, diabetes mellitus and malignant diseases are in line with that in the literature [11]–[16], [30], [31]. These associations seem biologically plausible, since thyroid hormones affect every cell and consequently a large number of positive associations between thyroid dysfunction and other diseases have been suggested. However, based on the existing literature, which is hampered by inadequate power and divergent findings, it is impossible to draw any firm conclusions as for the temporal relation between hypothyroidism and various morbidities [16].

Preceding, as well as following, the diagnosis of hypothyroidism there was an increased risk of being diagnosed with cardiovascular diseases, lung diseases, and diabetes mellitus. An explanation for this excess risk of a diagnosis of other diseases before the diagnosis of hypothyroidism could, at least for cardiovascular diseases, be that patients are at a higher risk of being diagnosed with hypothyroidism, due to treatment with e.g. iodine containing drugs (e.g. amiodarone) or exposure to contrast media [32]. On the other hand, the presence of morbidity before as well as after the diagnosis of hypothyroidism could also pertain to common environmental factors, such as smoking, related not only to development of hypothyroidism, but also to cardiovascular diseases, and lung diseases [33]. Also smoking cessation has been linked with the onset of hypothyroidism [34]. Our results could be in line with this, since patients suffering from e.g. chronic obstructive pulmonary disease have been found motivated for smoking cessation [35], which may transiently increase the risk of hypothyroidism [34]. Unfortunately, we can only speculate on the impact of smoking, since we had no access to smoking data. Besides the shared environmental factors, other possible explanations for finding increased morbidity, both preceding and following the diagnosis of hypothyroidism, could be shared genetic factors. There is a considerable genetic component in the etiology of hypothyroidism, diabetes mellitus, type I as well as type II, cardiovascular diseases, and lung diseases, and they could potentially be co-inherited [2], [36]–[38]. However, when controlling for genetic confounding, by using the twin pairs discordant for hypothyroidism, the increased risk of being diagnosed with morbidity persisted. This indicates that the results from the singleton analyses did not seem to be influenced by shared genetic factors. Unfortunately, although our study was population-based, there were too few discordant twin pairs to allow meaningful further stratification for zygosity and type of morbidity.

As autoimmune disorders tend to coexist in the same individuals [39], the increased risk of being diagnosed with diabetes, both preceding and following the diagnosis of hypothyroidism, seems in agreement with the available literature [40], [41]. Despite this, we found no significant association between hypothyroidism and rheumatic diseases. However, this is not necessarily in conflict with the literature, although an increased prevalence of rheumatoid arthritis associated with thyroid dysfunction has been suggested [16]. In our definition of rheumatic diseases we include a wide range of rheumatic diseases, including osteoarthrosis, which is far more common than e.g. rheumatoid arthritis [16].

A possible bias in epidemiological research is the risk of confounding by indication. In this case it refers to an increased awareness of other diseases due to diagnosis and treatment of hypothyroidism. In order to minimize this potential bias, and to ensure that the results were not confounded, we censored our morbidity data according to a time frame of 365 days before and after the diagnosis of hypothyroidism, and re-analyzed our findings. We cannot be certain that this procedure eliminates this kind of bias. However, the fact that our data did not change significantly, whether analyzed one way or the other, suggests that confounding by indication may not be a major issue. Moreover, in a historical cohort study, as the present study, selection bias could very well be present. In order to minimize this, the cases and controls were matched according to the principles of density sampling.

The strengths of our study include ascertainment of participants from nationwide population-based registers, high power due to large sample size, long observation period, and the use of validated and standardized procedures for evaluating the degree of morbidity [27], [42]. The possibility of stratifying for the time period before and after the diagnosis of hypothyroidism is unique to the present study, and allows raising the question of causality between hypothyroidism and any outcome of interest, such as morbidity. The fact that patients from both a hospital setting, whether in- or out-patient, and from primary care are accounted for minimizes the risk of selection bias. In contrast, although no systematic bias was introduced, the lack of information regarding the cause of hypothyroidism, as well as any effect of treatment on thyroid dysfunction, in line with most other surveys, are weaknesses. Also, the magnitude and effect of the potential bias caused by a possible change in help seeking behavior from the beginning to the end of the study is unknown. Moreover, our definition of disease groups may be oversimplified, and consequently the detection of morbidities incomplete.

In summary, hypothyroidism is significantly associated with other diseases. Prior to the diagnosis of hypothyroidism there is a significantly increased risk of being diagnosed with cardiovascular diseases, lung diseases, diabetes mellitus, and malignant diseases. Following the diagnosis of hypothyroidism there is a significant excess risk of being diagnosed with cardiovascular diseases, lung diseases, and diabetes mellitus. Our data suggest that neither genetic confounding nor confounding by indication constitute major pitfalls in our interpretation.
